# Differentially regulated genes in *Esr2*-mutant rat granulosa cells

**DOI:** 10.1016/j.dib.2018.05.098

**Published:** 2018-05-31

**Authors:** Vincentaben Khristi, V. Praveen Chakravarthi, Prabhakar Singh, Subhra Ghosh, Archit Pramanik, Anamika Ratri, Shaon Borosha, Katherine F. Roby, Michael W. Wolfe, M.A. Karim Rumi

**Affiliations:** a Department of Pathology and Laboratory Medicine, University of Kansas Medical Center, Kansas City, KS 66160, United States; b Department of Molecular and Integrative Physiology, University of Kansas Medical Center, Kansas City, KS 66160, United States; c Department of Anatomy and Cell Biology, University of Kansas Medical Center, Kansas City, KS 66160, United States; d Institute for Reproductive Health and Regenerative Medicine, University of Kansas Medical Center, Kansas City, KS 66160, United States

## Abstract

RNA seq analyses were performed in granulosa cells (GCs) collected from gonadotropin treated ESR2 mutant rats. Data obtained from a null mutant with *Esr2* exon 3 deletion (∆3) and another DNA binding domain (DBD) mutant with exon 4 deletion (∆4) were compared to that of wildtype (WT) rats. The raw data were analyzed using CLC genomics workbench. High quality RNA-sequencing reads were aligned to the *Rattus norvegicus* genome. Differentially expressed genes in ∆3 or ∆4 *Esr2*-mutant GCs were identified based on the following criteria: FDR p-Value ≤0.05 and an absolute fold change of 2. Fewer differentially expressed genes were identified in ∆3 compared to the ∆4 mutant group. As both mutant groups demonstrated a common phenotype of ovulation failure, differentially expressed genes common to both in ∆3 and ∆4 mutant rats were emphasized and further analyzed in the companion article “ESR2 regulates granulosa cell genes essential for follicle maturation and ovulation” [1].

**Specifications Table**

**Table:** 

Subject area	*Biology*
More specific subject area	*Reproductive biology*
Type of data	*RNA-Sequencing data table in Excel format, and figure*
How data was acquired	*Sequencing of RNA from granulosa cells*
Data format	*Normalized, analyzed and filtered data*
Experimental factors	*The four-week-old female rats were treated with exogenous gonadotropins*
Experimental features	*To identify the role of ESR2 in ovulation, ESR2 mutant (∆3 and (∆4)) and wild type rats were treated with 30 IU of PMSG. After 48 h of PMSG treatment, rats were injected with 30 IU hCG. Granulosa cells were collected 10 h after hCG treatment, RNA was isolated and analyzed by RNA sequencing. The differentially expressed genes were identified by CLC Genomic Workbench.*
Data source location	*Kansas City, KS 66160, USA*
Data accessibility	*Raw data has not yet been submitted to any public repository*

**Value of the data**

• This study provides the transcriptomic analyses of granulosa cells of ESR2 mutant rats (∆3 and ∆4).
• *Esr2*-mutant female rats from both groups were infertile due to failure of ovulation. Differentially expressed genes in *Esr2* mutant GCs may represent the ESR2 target genes downstream of gonadotropin induced ovulation.

## 1 Data

• Supplementary Table 1. Differentially expressed genes in gonadotropin stimulated Δ3 mutant granulosa cells.
• Supplementary Table 2. Differentially expressed genes in gonadotropin stimulated Δ4 mutant granulosa cells.
• Supplementary Table 3. Differentially expressed genes common to gonadotropin stimulated Δ3 and Δ4 mutant granulosa cells.
• Supplementary Table 4. Complete list of genes: Δ3 vs wild-type granulosa cells.
• Supplementary Table 5. Complete list of genes: Δ4 vs wild-type granulosa cells.

## 2 Experimental design, materials and methods

### 2.1 ESR2 mutant rats

All procedures were performed in accordance with the protocols approved by the University of Kansas Medical Center Animal Care and Use Committee. Holtzman Sprague-Dawley (HSD) *Esr2-*mutant rat models were generated by targeting exon 3 (∆3) or exon 4 (∆4) in the *Esr2* gene as described previously [2]. ∆3 caused a frameshift and null mutation in the ESR2 coding sequence while ∆4 resulted in an ESR2 protein lacking part of the DBD [2]. All animals were screened for mutation by PCR based genotyping using tail-tip DNA samples (RED extract-N-Amp Tissue PCR Kit, Sigma-Aldrich) and primers targeting the flanking intron sequences [2].

### 2.2 Treatment with exogenous gonadotropins

Four-week-old *Esr2*-mutant (∆3 and ∆4) and age-matched wildtype (WT) female rats were used for the gonadotropin induced follicular development. Synchronized follicular growth was initiated by intraperitoneal injection of 30 IU Pregnant Mare׳s Serum Gonadotropin (PMSG, National Hormone and Peptide Program). 48 h after the PMSG injection, 30 IU of Human Chorionic Gonadotropin (hCG, National Hormone and Peptide Program) was injected intraperitoneally.

### 2.3 Sample collection and processing

Animals were sacrificed 10 h after exogenous gonadotropin administration. GCs were collected from the gonadotropin treated WT, ∆3 and ∆4 ovaries, and total RNA was extracted by using TRI Reagent (Millipore-Sigma) following the manufactures instruction. RNA quantification was performed by using nanodrop (Thermo Scientific) and approximately 500 ng of total RNA was used for the RNA-seq library preparation. Libraries were prepared by using TruSeq standard mRNA kit (Illumina) following the manufacturer׳s instruction. The cDNA libraries were sequenced at the Molecular Biology Core Laboratory of Mayo Clinic (Rochester, MN).

### 2.4 RNA-seq data analyses

RNA-Seq data were analyzed by using the CLC Genomics Workbench (Qiagen Bioinformatics) to identify the differentially expressed genes. All clean reads were obtained by removing low quality reads by trimming, and the high-quality reads were aligned to the *Rattus norvegicus* genome (downloaded from NCBI database) using default parameters: (a) maximum number of allowable mismatches was 2 (b) minimum length and similarity fraction was set at 0.8; and (c) minimum number of hits per read was 10. A total of 32,623 genes were detected in each group of GCs. Expression values were measured in RPKM (Reads per kilobase of exon model per million mapped reads) [3]. The threshold p-value was determined according to the false discovery rate (FDR). In this study, genes that were considered differentially regulated met the following criteria: FDR p-value ≤0.05 and absolute fold change was 2.

## 3 Statistical analysis

For RNA Seq, each study group contained three library samples. Each library sample was made by pooling two RNA samples from two individual rats from the same genotype. In CLC Genomics Workbench, the ‘Differential Expression for RNA-Seq tool’ performs some multi-factorial statistics on a set of Expression Tracks based on a negative binomial Generalized Linear Model (GLM). The final GLM fit and dispersion estimate calculate the total likelihood of the model given the data, and the uncertainty on each fitted coefficient. Two statistical tests- Wald test and Likelihood Ratio test, each make use of one of these values. The Likelihood Ratio test is used in the Across groups (ANOVA-like) comparison.

## Appendix A Supplementary material

Supplementary Table 1. Differentially expressed genes in gonadotropin stimulated Δ3 mutant granulosa cells.

Supplementary Table 2. Differentially expressed genes in gonadotropin stimulated Δ4 mutant granulosa cells.

Supplementary Table 3. Differentially expressed genes common to gonadotropin stimulated Δ3 and Δ4 mutant granulosa cells.

Supplementary Table 4. Complete list of genes: Δ3 vs wild-type granulosa cells.

Supplementary Table 5. Complete list of genes: Δ4 vs wild-type granulosa cells.

## Footnotes

Appendix A Supplementary data associated with this article can be found in the online version at doi:10.1016/j.dib.2018.05.098.
