# Supplementary material for: Differentially regulated genes in Esr2-mutant rat granulosa cells
Source: Data Brief. 2018 May 31;19:1008–11. doi: 10.1016/j.dib.2018.05.098 (PMC5997925; doi:10.1016/j.dib.2018.05.098)
Supplement: Supplementary file 1 — Supplementary material [file mmc1.docx]

The authors do not have any conflict of interest.
